# Comparison of Conventional and Virtual Non-contrast Abdominal Images Using the Third-Generation Dual-Source Dual-Energy Computed Tomography

**DOI:** 10.7759/cureus.70017

**Published:** 2024-09-23

**Authors:** Ali Can Yalçın, Gonca Erbas

**Affiliations:** 1 Radiology, Gazi University Faculty of Medicine, Ankara, TUR

**Keywords:** abdominal imaging, dual-energy ct, dual source ct, virtual non contrast, virtual unenhanced

## Abstract

Purpose: To determine the efficacy and safety of virtual unenhanced imaging by comparing the attenuation values of virtual and true unenhanced images acquired using third-generation dual-source dual-energy computed tomography (dsDECT).

Methods: Single-energy non-contrast and dual-energy arterial and venous phase images of 97 patients who underwent triphasic abdominal computed tomography (CT) were included in this retrospective study. Virtual unenhanced images were generated for the arterial (a) and venous (v) phases using two dsDECT algorithms. The attenuation values were measured on the true and virtual unenhanced images of the liver, spleen, kidney, gallbladder, paraspinal muscle, aorta, subcutaneous fat, retroperitoneal fat, and renal cysts.

Results: A statistically significant difference was observed between the attenuation values ​​of true and virtual unenhanced images for all tissues (p < 0.001-0.025), except the venous phase virtual unenhanced images of the kidney, renal cysts, and gallbladder (p = 0.061-0.325). The proportion of cases with differences of ≥ 10 Hounsfield unit (HU) in the attenuation values between the virtual and true unenhanced images ranged from 3% to 8% for renal parenchyma, renal cysts, and gallbladder using this algorithm; however, this proportion was up to 90% for adipose tissue. No significant correlation was observed between the body mass index and attenuation differences between the true and virtual unenhanced images, except for those of the aorta and paraspinal muscle.

Conclusion: Virtual unenhanced images acquired using third-generation dsDECT cannot replace true unenhanced images in clinical practice owing to the difference between the attenuation values and variability of attenuation between true and virtual unenhanced images.

## Introduction

Dual-energy computed tomography (DECT) emits X-rays at two different energy levels and achieves material separation using the attenuation differences between tissues. DECT classifies voxels according to their elemental density by scanning them with X-rays emitted at two different energy levels and measuring the attenuation difference [1-4]. DECT facilitates material separation, which enables the acquisition of virtual non-contrast (VNC) images from contrast-enhanced images without additional examination.

Abdominal CT examinations characterize masses detected in solid organs, determine the aetiology of hematuria, evaluate endovascular stents, identify sources of gastrointestinal bleeding, and identify lesions detected incidentally during an examination. Attenuation values measured on non-contrast images provide insights into the contrast enhancement of lesions and aid in visualizing active contrast extravasation and calcifications [5]. DECT facilitates VNC image acquisition via the subtraction of iodine from contrast-enhanced images. Moreover, replacing true non-contrast (TNC) images with VNC datasets obtained from DECT can significantly reduce radiation exposure [6-8].

Previous studies investigated the effectiveness of VNC images; however, studies on the utility and efficacy of third-generation dual-source DECT (dsDECT) devices are limited. Consequently, there is insufficient information on the effectiveness of VNC imaging in the obese population [9].

Thus, this study aimed to determine the clinical effectiveness and reliability of VNC images acquired using dsDECT with two different algorithms by comparing the attenuation values of the VNC images with those of TNC images. This study also investigated the effect of obesity on the reliability of VNC imaging.

## Materials and methods

Study design

This retrospective study was approved by the Institutional Review Board and was conducted in compliance with the principles outlined in the Declaration of Helsinki. Informed consent was obtained from the patients. Contrast-enhanced CT images acquired in the same phases for cancer staging or follow-up, as requested by oncologists, of 101 patients who underwent multiphasic contrast-enhanced CT with a dsDECT device between January 2018 and January 2019 were retrospectively analyzed. Four patients were excluded from the study: two with intense motion artefacts and two with metallic artefacts caused by lumbar metallic fixation materials. Thus, 97 patients were included in the final analysis.

The height and weight of each patient were recorded to calculate the body mass index (BMI). Ages and radiation doses (computed tomography dose index (CTDI) and dose length product (DLP)) received during the examination were also recorded.

CT protocol

CT examinations were performed using SOMATOM Force (Siemens Healthcare, Forchheim, Germany), a third-generation dsDECT device. All patients underwent CT examinations using a triphasic CT protocol, including non-contrast, arterial, and venous phases, which is our department's standard for oncological evaluation.

TNC images covering the upper abdomen, extending from the liver dome to the inferior kidney, were acquired subsequently in the single energy mode. Acquisition parameters were as follows: detector collimation, 0.6 mm; tube rotation time, 0.5 s; and pitch, 0.6. The tube current and potential were set as 120 Quality Reference mA (Siemens Healthcare) and 120 kVp, respectively.

An 18-20G venous cannula was placed in the antecubital fossa of each patient, and 0.5 gI/kg of iodinated contrast medium (Optiray 350/100 mL, Guerbet, Liebel-Flarsheim, Canada) was administered at a rate of 4 mL/s using an automatic injector (MEDRAD Stellant Bayer HealthCare, Pennsylvania, USA). Cannula was flushed at the same rate using 30 mL of 0.9% NaCl subsequently. The Bolus tracking method was applied for contrast-enhanced imaging. Region of interest (ROI) was placed on the abdominal aorta at the level of the L1 vertebra, and the threshold value was set as 100 HU. Arterial and venous phase scanning was initiated at 15 and 65-70 s, respectively, after reaching the threshold.

Arterial-phase images covering the upper abdomen from the liver dome to the lower border of the kidney and venous-phase images covering the entire abdomen from the upper border of the liver to the inferior ischial bones were acquired. Contrast images were acquired using the dual-energy mode. Acquisition parameters were as follows: detector collimation, 0.6 mm; tube rotation time, 0.5 s; and pitch, 0.5. Tube current and potential were set to 100 kVp and 190 Quality Reference mAs, respectively, for tube A, and 150 kVp and 95 Quality Reference mAs, respectively, for tube B, following the manufacturer’s instructions. Automatic tube current modulation was applied to both tubes in the open position.

A soft tissue kernel (Br40, Siemens Healthcare) with a slice thickness and slice range of 1.5 mm and 0.75 mm, respectively, was used to reconstruct the TNC images. A soft tissue kernel (Qr40; Siemens Healthcare) with a slice thickness and slice range of 1.5 mm and 0.75 mm, respectively, was used to reconstruct the dual-energy images. An iterative reconstruction technique (ADMIRE; Siemens Healthcare) was used in the second degree for both sets of images. Dual-energy images representing conventional single-energy CT images at 120 kVp were reconstructed by obtaining 60% and 40% of the information from A and B tubes, following the manufacturer’s instructions.

A special imaging platform (SyngoVia Version VB20A_HF05, Siemens Healthcare) was used to transform dual-energy contrast images into VNC images. Two commercial software packages (Virtual Unenhanced and Liver VNC; Siemens Healthcare) with a triple-material separation algorithm were used to construct the VNC images. VNC images were reconstructed with a section thickness and cross-section spacing of 1.5 mm and 0.75 mm, respectively. Images obtained in the arterial and venous phases were processed separately using the liver virtual non-contrast (LVNC) and virtual unenhanced (VUE) algorithms, respectively; thus, four sets of VNC images were obtained for each patient.

Evaluation of images

**Figure 1:**
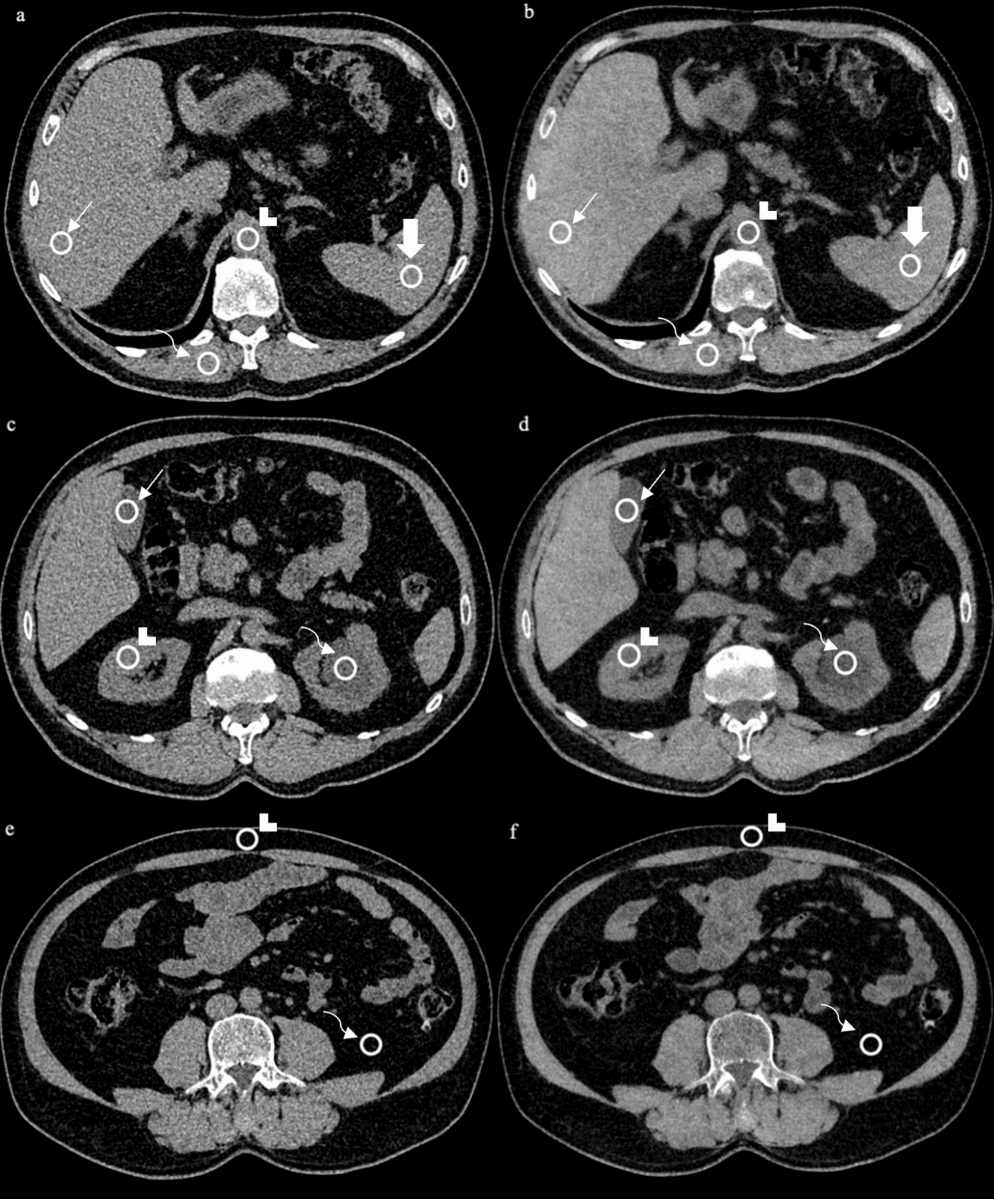
Example of measurement performed on virtual and true non-contrast images In the figure, “a, c, e” represent true non-contrast images, while “b, d, f” show virtual non-contrast images with measured attenuation samples. In figures “a, b”: liver (arrow), aorta (arrowhead), spleen (thick arrow), paraspinal muscle (curved arrow). In figures “c, d”: gallbladder (arrow), kidney (arrowhead), renal cyst (curved arrow). In figures “e, f”: subcutaneous fat (arrowhead), retroperitoneal fat (curved arrow).

The TNC, arterial phase (VUEa), venous phase (VUEv), arterial phase (LVNCa), and venous phase (LVNCv) images were created and saved in the Picture Archiving and Communication System (PACS) system (ExtremePACS Client, Version 4.3; Siemens Healthcare) of our hospital. Attenuation values of the liver, spleen, kidney parenchyma, gallbladder, paraspinal muscle, aorta, and subcutaneous and retroperitoneal adipose tissues were obtained from each image. A round ROI with an area of ​​100 mm^2^ was manually placed to measure the attenuation values. All series were opened side by side, and the attenuation values of the same section and area were obtained. Values were averaged to obtain the average Hounsfield Unit (HU) value. Examples of VNC images and attenuation measurements are given in Figure 1.

Statistical analysis

Statistical Package for the Social Sciences (IBM, SPSS Statistics for Macintosh, Version 25.0, Armonk, Newyork, USA) was used for data analysis. Descriptive data are presented as means± standard deviation, median, or percentage. The normality of the data distribution was evaluated using the Kolmogorov-Smirnov test before testing the hypotheses. Wilcoxon test analyzed the attenuation values of retroperitoneal and subcutaneous fat tissue, which did not show normal data distribution. All other normally distributed data were analyzed using the dependent-sample t-test. The attenuation values of the adipose tissue were evaluated using Spearman’s analysis. Attenuation values of the other tissues were evaluated using Pearson’s analysis to determine the correlation of attenuation differences between the true and VNC images with BMI and age. An r-value of <0.2, 0.2-0.4, 0.4-0.6, 0.6-0.8, and >0.8 indicated no, weak, moderate, high, and strong correlation, respectively. Bland-Altman analysis was performed for each VNC image and tissue to determine the variability of attenuation differences between the true and virtual images. A p-value of <0.05 was considered statistically significant.

## Results

Demographic and clinical data

Ninety-seven patients, comprising 43 men (45%) and 54 women (55%) with a mean age of 62.2±9.8 years (38-85 years), were included. The mean BMI of the patients was 28.7±5.3 (17.8-45.8). The BMI was <18.5, 18.5-24.9, 25-29.9, 30-34.9, 35-39.9, and ≥ 40 in one (1%, underweight), 27 (28%, normal weight), 37 (38%, overweight), 20 (21%, grade I obese), eight (8%, grade II obese), and four (4%, morbidly obese) patients, respectively.

The administered radiation dose was calculated using the CTDI (mGy) and DLP (mGycm). Average CTDI was 6.3±1.8 mGy (2.6-12.3), 7.9±2.4 (3.5-16.7) mGy, and 8.5±2.1 (4-15.5) mGy for the uncontrasted single-energy phase, dual-energy late arterial phase, and venous phase, respectively. Average DLP was 189±79 mGycm (63-436), 193±73 mGycm (77-455), and 415±120 mGycm (213-806) for the uncontrasted single-energy phase, and dual-energy late arterial phase, and venous phase, respectively. The total mean radiation dose was 829±250 mGycm (395-1583 mGycm). The estimated DLP obtained by excluding the TNC images from the acquisition protocol was 22.5%±5.9% (21.3-23.7, p<0.001).

Comparison of the attenuation values ​​of the TNC and VNC images

Attenuation values of the gallbladder could not be obtained in 32 patients, among whom 19 underwent gallbladder surgery, and 13 had a contracted gallbladder. Attenuation values of the subcutaneous adipose tissue could not be obtained in two patients. Attenuation values of the retroperitoneal adipose tissue attenuation measurements could not be obtained in three patients owing to the low amount of adipose tissue.

**Figure 2:**
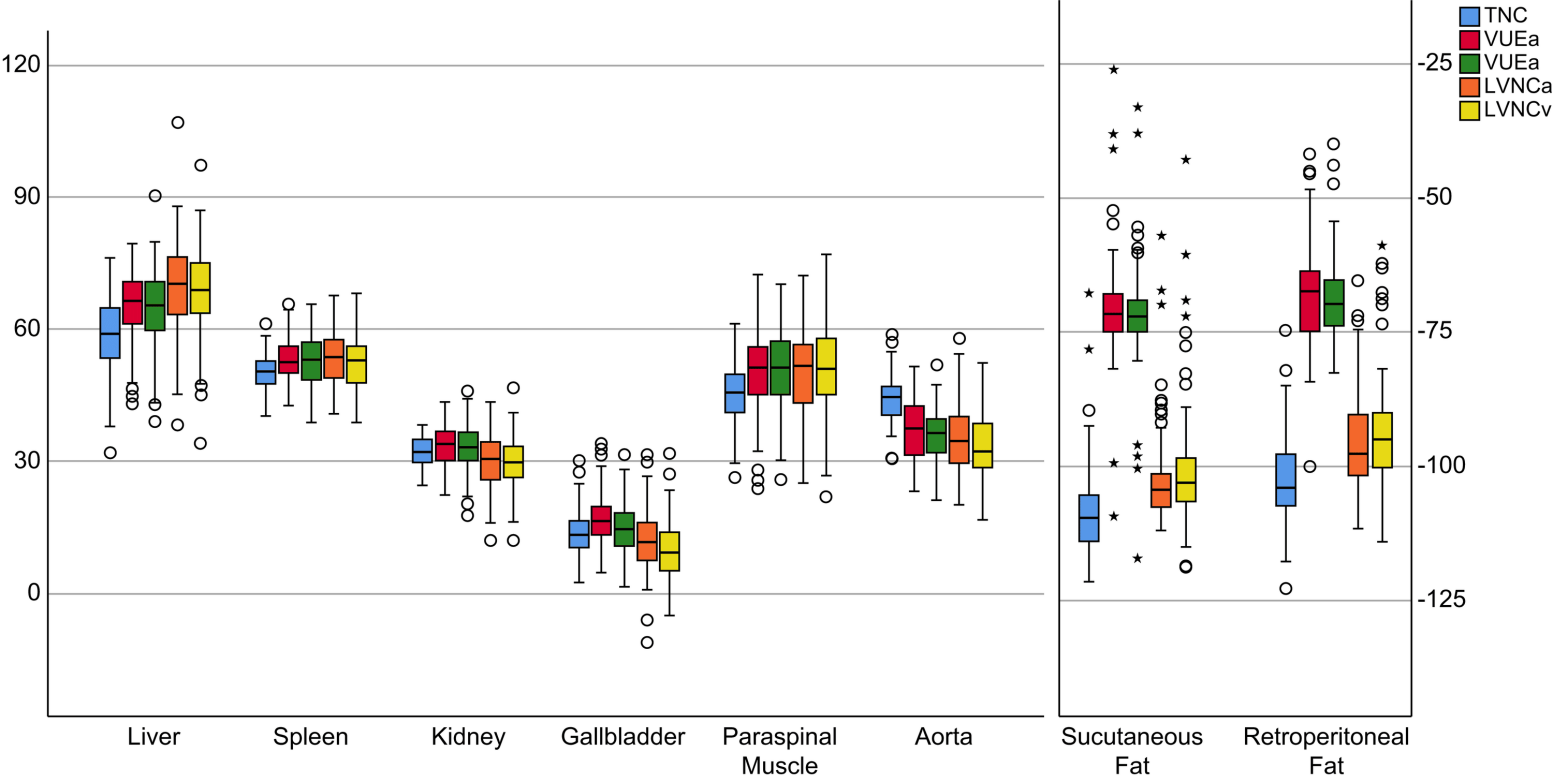
Box-and-whisker plots The boxes present the interquartile range (25-75%), the lines in the middle of the boxes represent the mean values, and the straight vertical lines represent the attenuation distribution range. Points marked with a circle (o) represent extreme values, whereas points marked with an asterisk (*) represent excessive values. TNC: true non-contrast; VUE, virtual unenhanced; LVNC, liver virtual non-contrast; a: arterial phase; v: venous phase

No statistically significant difference was observed between the attenuation values ​​of the TNC and VNC images of the kidney (p=0.066) and gallbladder (p=0.061) among the VUEv images. A statistically significant difference was observed between the attenuation values ​​of the TNC and VNC images of all tissues (p <0.001-0.025). Table 1 compares the attenuation values ​​of the TNC and VNC images. Figure 2 presents the attenuation distributions.

**Table 1:** Comparison of the attenuation values on true and virtual unenhanced images HU: Hounsfield unit; SD: standard deviation; TNC: true non-contrast; VUE: virtual unenhanced; LVNC: liver virtual non-contrast; a: Arterial phase; v: venous phase

Tissue	TNC Mean HU (±SD)	VUEa			VUEv			LVNCa			LVNCv		
		Mean HU (±SD)	Mean HU Difference (±SD)	p-value	Mean HU (±SD)	Mean HU Difference (±SD)	p-value	Mean HU (±SD)	Mean HU Difference (±SD)	p-value	Mean HU (±SD)	Mean HU Difference (±SD)	p-value
Liver	58.5 (8.1)	65.2 (8)	6.6 (5.7)	<0.001	64.8 (8.8)	6.2 (6.6)	<0.001	68.6 (10.6)	9.9 (6.9)	<0.001	68.4 (10.1)	9.8 (7.3)	<0.001
Spleen	50.3 (3.8)	53.1 (4.9)	2.8 (6.3)	<0.001	52.6 (5.8)	2.3 (6.4)	0.001	53.6 (5.9)	3.3 (7.2)	<0.001	52.4 (6.1)	2.1 (6.9)	0.003
Kidney	32.3 (3.2)	33.8 (4.7)	1.6 (5)	0.002	33.3 (5.1)	1 (5.4)	0.061	30.3 (6)	2 (5.8)	0.001	29.6 (5.5)	2.7 (5.5)	<0.001
Gallbladder	13.9 (5.8)	17.5 (6.4)	3.5 (4.4)	<0.001	15.15 (6.5)	1.2 (4.9)	0.066	12.32 (7.8)	1.6 (5.8)	0.025	10.1 (7.1)	3.9 (5.9)	<0.001
Paraspinal Muscle	45.1 (6.9)	50.6 (9.4)	5.5 (7.1)	<0.001	50.2 (9.5)	5.1 (7.6)	<0.001	50.3 (10.6)	5.2 (7.6)	<0.001	50.8 (10.7)	5.7 (8.4)	<0.001
Aorta	44.2 (5.1)	37.4 (6.9)	6.7 (8.4)	<0.001	35.5 (5.8)	8.6 (7.3)	<0.001	35.2 (8.1)	8.9 (9.3)	<0.001	33.3 (7.3)	10.8 (8.6)	<0.001
Subcutaneous Fat	-108.2 (8.4)	-70.2 (10.1)	38.1 (9)	<0.001	-71.5 (10.5)	36.7 (10.4)	<0.001	-102.4 (8.8)	5.8 (5.9)	<0.001	-100.6 (11.2)	7.6 (9)	<0.001
Retroperitoneal Fat	-102.5 (8.2)	-68.8 (8.9)	33.7 (8.5)	<0.001	-68.9 (7.8)	33.5 (8.6)	<0.001	-95.5 (9.1)	6.9 (6.5)	<0.001	-93.9 (10.7)	8.6 (6.9)	<0.001
Renal Cyst	9.1 (3.6)	9.8 (4.8)	0.8 (4.9)	0.325	10.2 (5.3)	1.1 (4.9)	0.168	3.1 (4.1)	5.9 (3.8)	<0.001	3.5 (4.8)	5.9 (4.1)	<0.001

**Figure 3:**
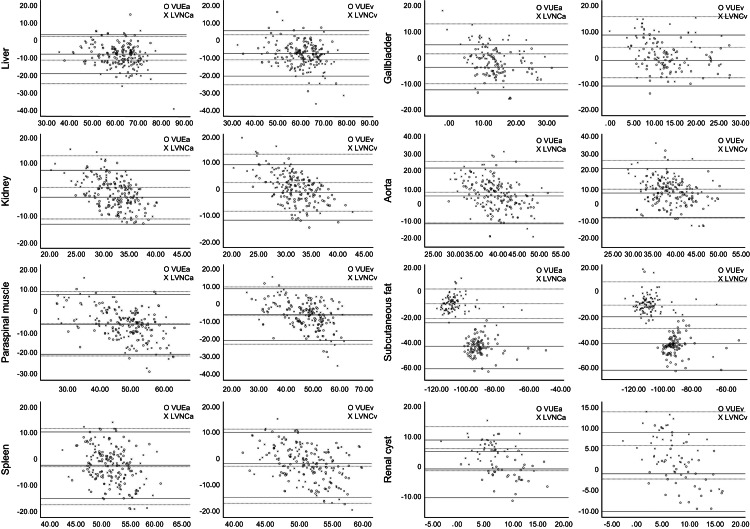
Bland-Altman plots The central line in the graph represents the mean difference value and the lines at the top and bottom represent 1.96xSD. The solid lines represent virtual unenhanced images (VUE), whereas the dashed lines represent liver virtual unenhanced images (LVNC). The points on the graph represent each measured data. a: arterial phase; v: venous phase

Evaluation of the tissue attenuation values of the VNC images revealed lower attenuation values ​in all VNC images (VUE and LVNC) of the aorta and the VNCa and VNCv images of the gallbladder and kidney compared with the TNC images. On average, higher attenuation values ​​were observed in the VNC images of all tissues. Table 2 presents a patient-based comparison of the attenuation measurements. Figure 3 presents the Bland-Altman graphs delineating the differences in attenuation.

**Table 2:** Numbers and percentages of patients with an attenuation difference of <5, 10, and 20 HU between the true and virtual non-contrast images HU: Hounsfield Unit; n: number of patients; VUE: virtual unenhanced; LVNC: liver virtual non-contrast; a: arterial phase; v: venous phase

Tissue	n	HU Difference	VUEa	VUEv	LVNCa	LVNCv
Liver		<5	34 (35.1%)	33 (34.0%)	17 (17.5%)	19 (19.6%)
	97	<10	69 (71.2%)	71 (73.2%)	45 (46.4%)	51 (52.6%)
		<20	96 (98.9%)	95 (97.9%)	91 (93.8%)	90 (92.8%)
Spleen		<5	48 (49.5%)	51 (52.6%)	43 (44.3%)	54 (55.7%)
	97	<10	83 (85.6%)	82 (84.6%)	75 (77.3%)	79 (81.5%)
		<20	97 (100%)	97 (100%)	97 (100%)	97 (100%)
Kidney		<5	68 (70.1%)	65 (67.0%)	54 (55.7%)	56 (57.7%)
	97	<10	91 (93.8%)	89 (91.7%)	90 (92.8%)	91 (95.9%)
		<20	97 (100%)	97 (100%)	97 (100%)	97 (100%)
Gallbladder		<5	37 (56.9%)	42 (64.6%)	39 (60%)	33 (50.8%)
	65	<10	62 (95.4%)	62 (95.4%)	59 (90.8%)	54 (83.1%)
		<20	65 (100%)	65 (100%)	65 (100%)	65 (100%)
Paraspinal Muscle		<5	40 (41.2%)	42 (43.3%)	43 (44.3%)	41 (42.3%)
	97	<10	74 (77.3%)	72 (74.2%)	75 (77.3%)	69 (71.2%)
		<20	94 (96.9%)	95 (97.9%)	94 (96.9%)	92 (94.8%)
Aorta		<5	31 (32.0%)	24 (24.7%)	24 (24.7%)	21 (21.6%)
	97	<10	61 (62.9%)	53 (54.6%)	46 (47.4%)	39 (40.2%)
		<20	90 (92.8%)	93 (95.9%)	87 (89.7%)	84 (86.6%)
Subcutaneous Fat	95	<10	2 (2.1%)	1 (1.1%)	74 (77.9%)	64 (67.4%)
		<20	3 (3.2%)	6 (6.4%)	93 (97.9%)	91 (95.8%)
		<30	11 (11.6%)	16 (16.9%)	95 (100%)	92 (96.9%)
		<40	54 (56.9%)	67 (70.6%)	-	93 (98%)
Retroperitoneal Fat	94	<10	1 (1.1%)	1 (1.1%)	70 (74.5%)	61 (64.9%)
		<20	6 (6.4%)	4 (4.3%)	89 (94.7%)	88 (93.6%)
		<30	22 (23.4%)	31 (33.0%)	94 (100%)	92 (97.9%)
		<40	77 (81.9%)	75 (79.8%)	-	94 (100%)
Renal Cyst	38	<5	27 (71.1%)	24 (63.2%)	14 (36.8%)	18 (47.4%)
		<10	36 (94.8%)	37 (97.4%)	33 (86.8%)	32 (84.2%)
		<20	38 (100%)	38 (100%)	38 (100%)	38 (100%)

Correlation analysis

Differences between the attenuation values of the paraspinal muscle measurements showed a statistically significant (p<0.001-0.006) but weak (r=0.280-0.351) positive correlation with BMI in all series. A statistically significant (p<0.001) correlation was observed between the aortic measurements and BMI in all series, with a moderate positive correlation (r = 0.414-0.480) observed in the VUEa, VNCa, and VNCv images and a weak positive correlation (r=0.366) observed in the VUEv images. No significant correlation was observed between the BMI and attenuation differences in other tissues or VNC images.

## Discussion

Studies that used second-generation dsDECT reported higher attenuation values in the VNC images than in the TNC images, possibly due to insufficient removal of iodine. However, limitations of this method are known such as insufficient iodine removal and small dual energy field of view (FOV) size in second-generation devices [10,11]. We used third-generation dsDECT and demonstrated that the mean attenuation difference between the TNC and VNC images was lower for parenchymal organs. Moreover, removing iodine from the contrast-enhanced images was performed with higher success than De Cecco et al.'s study, which used second-generation dsDECT [12].

De Cecco et al. evaluated VNC images acquired using the LVNC algorithm using second-generation dsDECT and revealed no statistically significant difference between the attenuation values ​​of VNC and TNC images for the liver [13]. However, unlike this previous study, a low-energy X-ray tube voltage of 100 kVp instead of 90 kVp was used. In addition, VNC images were constructed using the VUE and LVNC algorithms. Contrary to De Cecco et al.'s study, a statistically significant difference was observed between the VNC imaging algorithms and TNC images; however, the difference between the attenuation of the TNC and VNC images was smaller in the liver compared with that observed in the studies performed with second-generation dsDECT. Furthermore, the mean attenuation difference for the LVNC algorithm (9.8-9.9 HU for LVNCa-LVNCv) was higher than the VUE algorithm (5.7-6.6 HU for VUEa-VUEv), which was developed particularly for liver assessment.

An attenuation difference of more than 15-20 HU between contrast-enhanced and non-contrast images enhances the contrast of the renal lesion and may indicate a neoplastic lesion; however, a limitation has not been defined in the literature [14,15]. A comparison of the attenuation measurements of the kidney parenchyma revealed no significant differences between the attenuation values of the VUEv and TNC images (p=0.061). Statistically significant differences were observed between the attenuation values of the TNC and VNC images in other VNC image series; however, the mean attenuation differences were <3 HU, which can be neglected in clinical practice. Evaluation of the mean and patient-based individual differences in the attenuation revealed that the attenuation values obtained using the VUE algorithm were closer to those of the TNC images than those obtained using the LVNC algorithm. Previous studies that investigated the role of VNC images in renal lesion characterization have reported that the effectiveness of VNC images may be reduced owing to variability in the attenuation difference between the TNC and VNC images. Thus, these studies recommend using VNC images with caution. However, VNC images yield diagnostically acceptable results close to those of TNC images [16,17].

Previous studies that investigated the role of DECT in the characterization of renal lesions, including simple renal cysts, showed that VNC images possess sufficient diagnostic efficiency for identifying these lesions; however, the basal attenuation values ​​of the lesions varied between the TNC and VNC images [18,19]. The attenuation values ​​of the VUE images were closer to those of TNC images than those of the LVNC images of renal cysts in the present study. An attenuation difference of 10-20 HU was detected in a small number of lesions (2.6-5.2%) on VUE images; however, the attenuation difference was <10 HU in a substantial number of patients (94.8-97.4%). This finding indicates that images constructed using the VUE method can replace TNC images. However, complex cystic lesions were not included in the present study, and no pathological correlation was observed.

A statistically significant difference was observed between the attenuation measurements of the TNC and VNC images for subcutaneous and retroperitoneal adipose tissues. Examination of the mean attenuation differences between the TNC and VNC images revealed that the results obtained using the LVNC algorithm were closer to those of the TNC images than those acquired using the VUE algorithm. LVNC may be more efficient than VUE among the two VNC imaging algorithms for fatty tissues investigated in this study. A sufficient number of patients with adrenal adenoma were not included in the present study; however, the interpretation of the measurements of the fatty tissue suggests that VNC imaging cannot replace TNC imaging for the differential diagnosis of fatty lesions, such as fat-rich adenomas, owing to the variability in the attenuation difference. These findings support previous studies wherein higher attenuation values ​​were observed in the VNC images of fat-rich adenomas [20,21].

Attenuation values for the aorta and kidney in all VNC images were lower than those in the TNC images. However, the attenuation values in all tissues and organs in all VNC images were higher than in the TNC images. Iodine may have been insufficiently or excessively removed from tissues and the aorta using VNC imaging algorithms that create images without contrast from contrast-enhanced examinations. Thus, the attenuation difference in the VNC images can be attributed to insufficient iodine extraction, the nature of the VNC imaging algorithms, and the software.

Durieux et al. used a similar model with a third-generation dsDECT device, and a statistically significant correlation between the attenuation difference between the TNC and VNC images and BMI in all tissues was observed [9]. The mean BMI in the present study was higher than that in the aforementioned study, and most patients included in the present study were overweight or obese. No significant correlation was observed between the BMI and attenuation differences in the parenchymal organs in the present study, contrary to Durieux et al.'s study.

All parenchymal organs in all patients, except one, were in the dual-energy FOV. A small section of the left kidney and spleen was outside the FOV owing to incorrect FOV positioning. However, it was determined that all parenchymal organs would remain within the FOV with correct FOV positioning. A previous study in a population with a mean BMI of 25 using second-generation dsDECT reported that the dual-energy FOV in the liver could not achieve full coverage, and a section of the liver remained outside the FOV in approximately 25% of the cases. This study demonstrated that third-generation dsDECT with a dual-energy FOV is adequately wide with correct patient positioning, even in individuals with morbid obesity.

DECT examinations do not increase the radiation dose compared with single-energy CT [10,22]. De Cecco et al. conducted a similar study with third-generation dsDECT and reported a dose reduction of approximately 32% after removing the TNC images from the protocol [13]. A radiation dose reduction of approximately 23% could be achieved by removing TNC images from the imaging protocol, as the images acquired in the venous phase included the entire abdomen. These data show that DECT and VNC imaging may be preferred for routine use owing to the significant reduction in radiation exposure and its contribution to diagnosis.

This study has some limitations. This was a retrospective study with a small sample size. Moreover, only quantitative variables were evaluated in this study. Further, the ROI position differed slightly between the VNC and TNC series as the ROIs were placed manually; this may have led to variations in the attenuation values. Furthermore, a single observer performed a single measurement, and the intra- and inter-observer reproducibility of the measurements was not investigated.

## Conclusions

The performance of VUE images was similar to that of TNC images for the renal parenchyma and simple renal cysts. Thus, TNC images can be replaced with VNC images in kidney evaluation. Significant differences were observed between the attenuation values of the VNC and TNC images in all tissues and organs other than the kidney parenchyma and cysts. Thus, VNC images cannot completely replace TNC images in clinical practice. The findings of this study suggest that using the LVNC algorithm for fatty tissues and fat-containing lesions and the VUE algorithm for fluid-containing lesions yields more accurate results. Further studies targeting specific lesions that use both algorithms must be conducted in the future to validate these findings.

This study revealed no significant effect of BMI ​​on the difference between the attenuation values of VNC and TNC images of the parenchymal organs in the evaluated areas. The use of VNC images may significantly reduce the radiation dose. The findings of this study suggest that DECT may aid in reducing the radiation dose in CT examinations in the future.
